# Impact of iterative reconstruction with resolution recovery in myocardial perfusion SPECT: phantom and clinical studies

**DOI:** 10.1038/s41598-019-56097-4

**Published:** 2019-12-23

**Authors:** Koichi Okuda, Kenichi Nakajima, Hiroto Yoneyama, Takayuki Shibutani, Masahisa Onoguchi, Shinro Matsuo, Mitsumasa Hashimoto, Seigo Kinuya

**Affiliations:** 1 Department of Physics, Kanazawa Medical University, Uchinada, Japan; 2 Department of Functional Imaging and Artificial Intelligence, Kanazawa University, Kanazawa, Japan; 3 Department of Radiological Technology, Kanazawa University Hospital, Kanazawa, Japan; 4 Department of Quantum Medical Technology, Kanazawa University, Kanazawa, Japan; 5 Department of Nuclear Medicine, Kanazawa University Hospital, Kanazawa, Japan

**Keywords:** Ischaemia, Radionuclide imaging

## Abstract

The corrections of photon attenuation, scatter, and depth-dependent blurring improve image quality in myocardial perfusion single-photon emission computed tomography (SPECT) imaging; however, the combined corrections induce artifacts. Here, we present the single correction method of depth-dependent blurring and its impact for myocardial perfusion distribution in phantom and clinical studies. The phantom and clinical patient images were acquired with two conditions: circular and noncircular orbits of gamma cameras yielded constant and variable depth-dependent blurring, respectively. An iterative reconstruction with the correction method of depth-dependent was used to reconstruct the phantom and clinical patient images. We found that the single correction method improved the robustness of phantom images whether the images contained constant or variable depth-dependent blurring. The myocardial perfusion databases generated from 72 normal patients exhibited uniform perfusion distribution of whole myocardium. In summary, the single correction method of depth-dependent blurring with iterative reconstruction is helpful for myocardial perfusion SPECT.

## Introduction

Iterative reconstruction^1^, such as ordered-subset expectation maximization (OSEM)^2^, is an indispensable technology in single-photon emission computed tomography (SPECT) imaging to correct depth-dependent blurring^3–9^, photon attenuation^10^, and scatter^11^ in nuclear medicine^12–16^. The iterative reconstruction technology with resolution recovery (RR) algorithm improves the signal-to-noise ratio of counts in myocardial perfusion SPECT imaging (MPI), and is feasible for performing half-time or half-dose SPECT acquisition^3,4^.

With respect to attenuation correction (AC), scatter correction (SC), and RR algorithm, our previous study exhibited the optimal OSEM reconstruction condition incorporating these processing^16^. Although the AC improves photon attenuation in the body, artifacts induced by AC, such as apical thinning may occur in SPECT images^17–20^. Furthermore, since attenuation-corrected myocardial perfusion images differ greatly from uncorrected images, nuclear medicine physicians usually have to compare corrected and non-corrected images to diagnose myocardial ischemia and infarction in clinical practice^21,22^. Although normal databases of myocardial perfusion SPECT have been created in Japanese subjects by the Japanese Society of Nuclear Medicine working group^23–25^, they were mainly based on non-AC images, and effects of OSEM were not evaluated. Therefore, we hypothesized that OSEM with RR (OSEM_RR_) could aid the interpretation of MPI without artifacts, and OSEM_RR_ is also helpful in the reconstruction of projection images. A large number of studies have been published using OSEM_RR_ regarding the improvement of FHWM and signal-to-noise ratio in basic phantom studies and wall thickness of the myocardium in clinical studies^26,27^. However, the improvement of distribution of myocardial perfusion is crucial to determine ischemia or infarction of the myocardium in MPI. To best of our knowledge in OSEM_RR_ processing, the count distribution of the whole myocardium using a polar map has only been reported by Zoccarato *et al*. research group^14,15,28^. However, the group focused on the comparison of gamma camera differences and reconstruction algorithm differences using count distribution. Moreover, uniform myocardial perfusion distribution was not determined when the number of iterations was change. Our motivation for this research was to provide the uniform count distribution with less depth-dependent blurring in myocardial perfusion SPECT images.

The aim of this study was to confirm that the myocardial perfusion SPECT distribution derived from OSEM_RR_ in both phantom and clinical studies reduces the differences between circular and non-circular orbits compared to reconstruction with FBP. Consequently, we generated a normal database (NDB) for OSEM_RR_ derived from patients with a low likelihood of coronary artery disease (CAD) and evaluated myocardial perfusion distribution.

## Materials and Methods

### Cone-shape phantom

**Figure 1:**
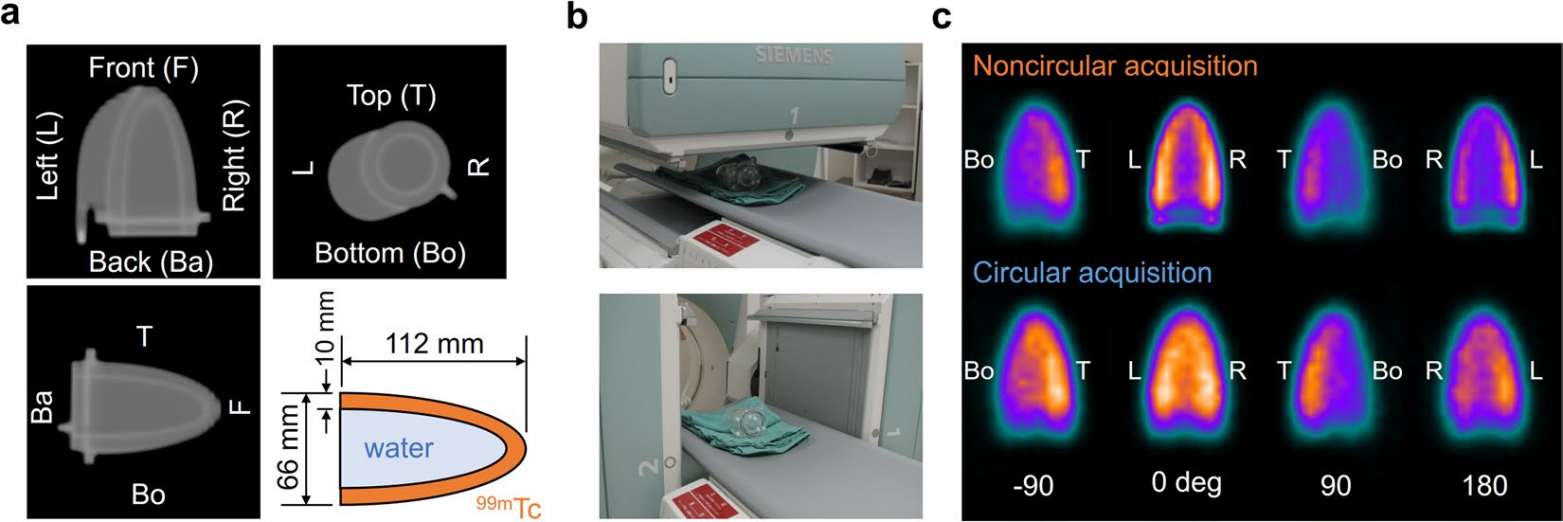
A cone-shape phantom. (**a**) A coronal image (upper left), a transaxial image (upper right), a sagittal image (bottom left) and the structure of the phantom (bottom right). (**b**) The positions of gamma cameras in the minimum (upper) and maximum (bottom) gaps between the phantom and gamma cameras in NCA. (**c**) The projection images derived from CA and NCA with the positions of gamma cameras at 0, ±90, and 180 degrees. *NCA*, noncircular acquisition; and *CA*, circular acquisition.

An acrylic cone-shape phantom has a height of 112 mm and a basal diameter of 66 mm (Fig. 1a). The compartment for the radioisotope was filled with 64 MBq of ^99m^Tc-pertechnetate, and an inner cavity was filled with non-radioactive water. The volumes of compartments for radioisotope and water were 118 mL and 93 mL, respectively. The noncircular orbit with the 360-degree rotation of gamma cameras was applied to the phantom acquisition in close proximity to the surface of the phantom and bed (Fig. 1b). A circular orbit was also applied in this phantom study.

### Anthropomorphic torso phantom

We utilized the anthropomorphic torso phantom configured with the cardiac, pulmonary and hepatic components (Kyoto Kagaku, Kyoto, Japan). The left ventricular (LV) myocardium and liver were filled with 185 and 19 MBq of Tc-99m pertechnetate, respectively. The left and right ventricular cavities were filled with non-radioactive water. The circular-orbit acquisition (CA) and noncircular-orbit acquisition (NCA) with the 360-degree rotation of gamma cameras were used for the anthropomorphic torso phantom study. Four plastic circular defects with a 20 mm diameter were placed in the mid-anterior, lateral, inferior, and septal walls.

### Study population

We retrospectively included 37 female and 35 male patients with a low likelihood of CAD from Kanazawa University Hospital. ^99m^Tc-sestamibi or ^99m^Tc-tetrofosmin MPI was performed with NCA of gamma cameras. The mean age of male and female patients was 68 ± 16 and 67 ± 15 years, respectively (p = n. s.). The mean height and weight were 151 ± 5 cm and 50 ± 6 kg for females, and 165 ± 8 cm and 66 ± 15 kg for males, respectively (p < 0.0001 for both). Approval for this study was obtained from the ethics committee of the Kanazawa University. The current study was performed in accordance with the ethical guidelines of the Declaration of Helsinki. Due to our retrospective study, informed consent was obtained from all subjects in the form of opt-out. Patients who rejected participation in our study were excluded.

### Image acquisition and data processing

In both phantoms and clinical studies, SPECT acquisition was performed with a dual-head gamma camera (Symbia T6 hybrid SPECT/CT scanner, Siemens Healthcare K.K., Tokyo, Japan) equipped with a low-energy high-resolution collimator. The pixel size was 6.6 mm for a 64 × 64 matrix. A photopeak energy window of ^99m^Tc was set at 15% centered at 140 keV. In the phantom study, acquisition time was set as 5 seconds per projection. 60 projection images with CA and NCA of gamma cameras were obtained. In the clinical study, we performed stress and rest gated MPI with 16 frames per cardiac cycle on the hybrid SPECT/CT scanner. MPI was performed with a 360-degree NCA with 60 projections at 40 minutes after injection of ^99m^Tc tracers of 300–370 MBq. The acquisition time was set as 35 seconds per projection.

### Data analysis

We used two-reconstruction processing methods: OSEM_RR_ and filtered back projection (FBP) in the phantom and clinical studies. The resolution recovery processing is based on a Flash 3D algorithm (Siemens Healthcare K.K., Tokyo, Japan)^29^. The Flash 3D algorithm was not incorporated into the FBP processing. In the phantom study, when the subset was constantly set as 15, the number of iterations was set at 1, 5, 10, 15, and 20. In the clinical study, subsets and iterations were set as 15 and 8, respectively. We utilized a Gaussian post-filter for OSEM_RR_ and a Butterworth filter for FBP in both studies. The full width at half maximum of the Gaussian filter was 13.2 mm. The cutoff frequency and order for the Butterworth filter were 0.68 Nyquist and 8. All OSEM_RR_ and FBP processing was performed using e.soft version 8.1 (Siemens Healthcare K.K., Tokyo, Japan).

In the cone-shape phantom and anthropomorphic torso phantom studies, circumferential profile analysis was used to assess SPECT count profiles^30,31^. The SPECT count profile was presented on a line chart as a function of angle, the origin of which corresponds to the 12 o’clock position on the phantom images. The angles were measured in a clockwise direction relative to an origin at 12 o’clock. The anterior, lateral, inferior, and septal regions of myocardium were defined as 315–45°, 45–135°, 135–225°, and 225–315° in the anthropomorphic torso phantom, respectively. The defect regions of myocardium were also defined as 345–15° for the anterior wall, 75–105° for the lateral wall, 165–195° for the inferior wall, and 255–285° for the septal wall in the anthropomorphic torso phantom with defect, respectively. The SPECT count profiles were evaluated in the central transaxial slice of the cone phantom and the central short axial slice of the anthropomorphic torso phantom. A count ratio of anterolateral to the anteroseptal region was calculated as segments 8 and 14 divided by segments 12 and 16 in the quantitative analysis of the NDB using a standard 17-segment model^32^. The count ratio of the anterolateral to the inferior region and that of the anterolateral to the anterior region were also calculated as segments 10 and 15 divided by segments 12 and 16, and segments 7 and 13 divided by segments 12 and 16, respectively. Polar maps were generated with quantitative perfusion SPECT (QPS) version 2008.1 (Cedars-Sinai Medical Center, Los Angeles, CA, USA).

The compatibility of Gaussian and Butterworth filters was evaluated in the anthropomorphic phantom study. When the cutoff frequency of Butterworth filter was set as 0.68 Nyquist in our clinical condition, the FWHM of Gaussian filter was changed from 3.3 to 16.5 mm. SPECT count profiles for the central short axial slice of the cardiac component were compared in both Gaussian and Butterworth filters. The comparative evaluation of OSEM_RR_ and FBP reconstructions with or without Gaussian filtering was also performed. SPECT count distributions were calculated for OSEM_RR_ and FBP images without Gaussian filtering, and a fast Fourier transform was used to convert the SPECT count distributions into the frequency domain using ImageJ version 1.52p (NIH, Bethesda, Maryland, USA).

### Statistical analyses

All continuous values were expressed as a mean ± standard deviation (SD). The differences in continuous variables were analyzed using Student’s t-test and Tukey-Kramer test. A paired t-test was used to analyze the differences in paired continuous data. All statistical tests were two-tailed, and a *p*-value of less than 0.05 was considered significant. These analyses were performed using the JMP version 11.2.1 (SAS Institute Inc., Cary, NC, USA) software.

## Results

### Cone-shape phantom

**Figure 2:**
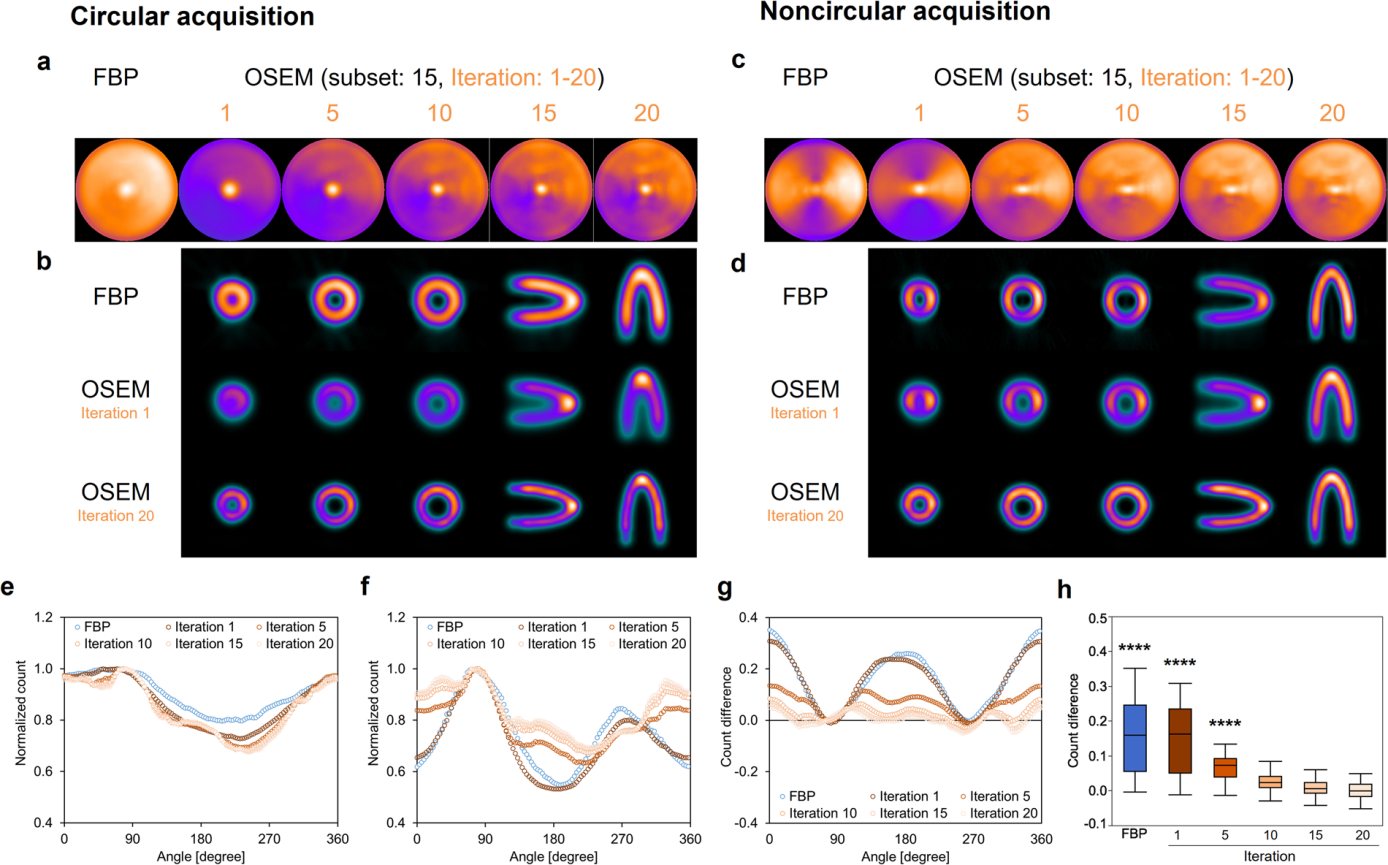
The cone phantom images and its count distributions. (**a**,**c**) Polar maps were generated with FBP and OSEM_RR_ reconstructions in CA and NCA. The iterations changed from 1 to 20, and a subset was constantly 15 in OSEM_RR_. (**b**,**d**) Transaxial images in the front, central, and base, sagittal images, and coronal images of the cone phantom in CA and NCA. (**e**) Normalized count distributions of the central transaxial slice in CA. (**f**) Normalized count distributions of the central transaxial slice in NCA. (**g**) Count differences between CA and NCA. (**h**) The box-and-whisker plot of the count differences. The count differences obtained from FBP and OSEM_RR_ with 1, 5, 10, and 15 iterations were compared with OSEM_RR_ with 20 iterations. (****P < 0.0001, Tukey-Kramer test). Error bars are SD of the mean. Abbreviations as in Fig. 1.

Figure 1 shows a cone-shape phantom and experimental condition. The projection images were obtained from NCA (Fig. 1b) and CA of gamma cameras. The rotation radius was constantly 230 mm in CA, and minimum and maximum radii were 91 mm and 230 mm in NCA, respectively. The superior resolution of projection images was visually shown at 0 and 180 degrees of gamma camera in NCA compared with CA (Fig. 1c). Acquired counts of the projection data ranged from 111 kilocounts/view at 91 mm to 62 kilocounts/view at 230 mm in NCA. The projection data acquired with circular and noncircular orbits of gamma cameras were reconstructed with FBP and OSEM_RR_ (Fig. 2). Polar maps derived from CA and NCA were different greatly in FBP (Fig. 2a,c). Polar maps derived from CA and NCA were visually similar in OSEM_RR_ with 10, 15, and 20 iterations except for the apex. Normalized SPECT count at the front region was higher in NCA than in CA in Supplementary Fig S1. An inner space of the cone phantom derived FBP and OSEM_RR_ with one iteration was smaller in CA than in NCA (Fig. 2b,d). That derived from OSEM_RR_ with 20 iterations was equivalent in CA and NCA. OSEM_RR_ with 5, 10, 15, and 20 iterations yielded similar normalized count profiles in CA, and that with 10, 15, and 20 iterations also yielded similar normalized count profiles in NCA (Fig. 2e,f). The largest and smallest normalized count differences were exhibited in FBP (0.16 ± 0.11) and OSEM_RR_ with 20 iterations (0.0026 ± 0.0059), respectively (*p* < 0.0001, Fig. 2g,h).

### Anthropomorphic torso phantom: normal myocardium model

**Figure 3:**
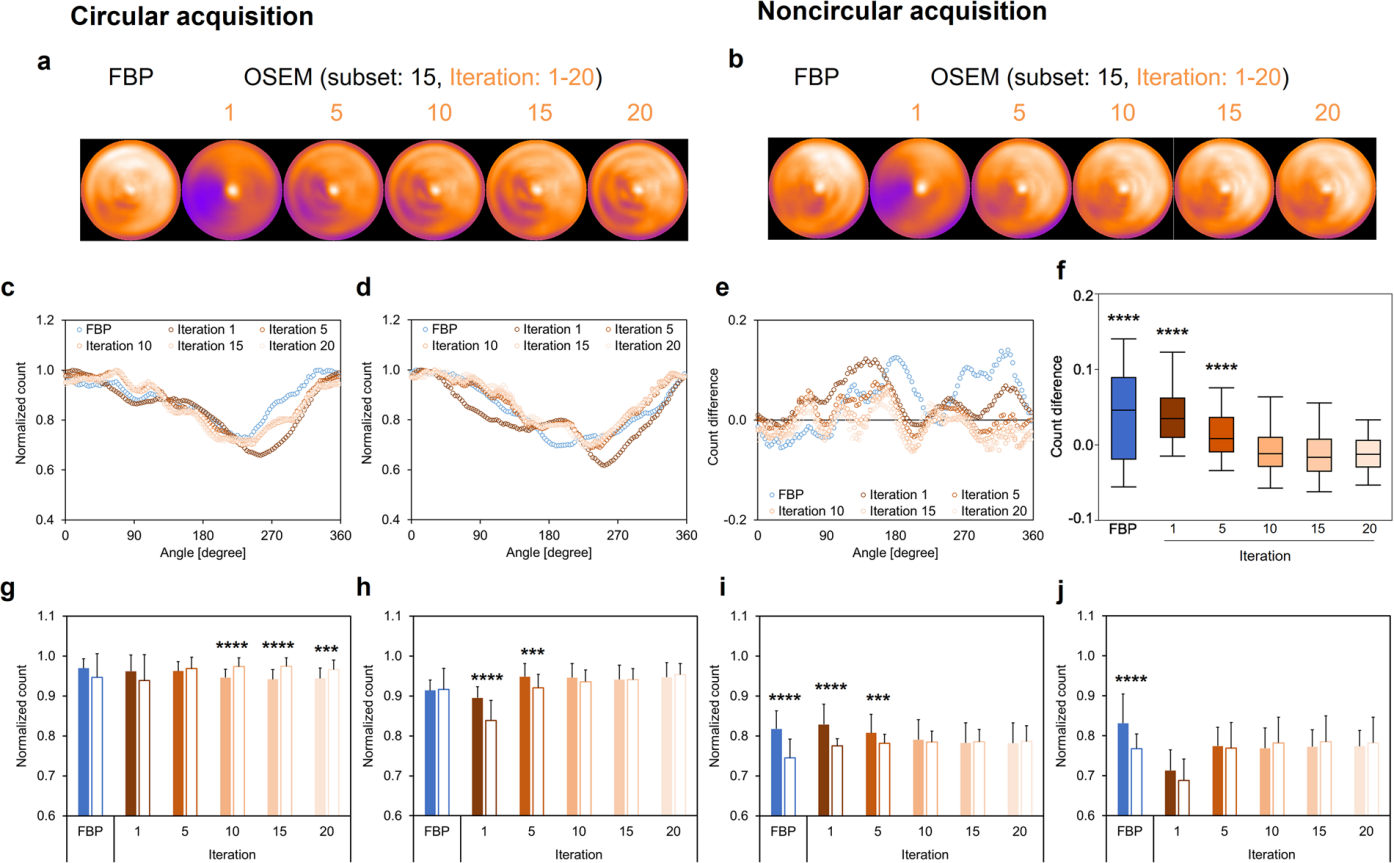
The anthropomorphic torso phantom images and its count distributions. Polar maps were generated with FBP and OSEM_RR_ reconstructions in CA (**a**) and NCA (**b**). The iterations changed from 1 to 20, and a subset was constantly 15 in OSEM_RR_. (**c**) Normalized count distributions of the short axis image in CA. (**d**) Normalized count distributions of the short axis image in NCA. (**e**) Count differences between CA and NCA. (**f**) The box-and-whisker plot of the count differences. The count differences obtained from FBP and OSEM_RR_ with 1, 5, 10, and 15 iterations were compared with OSEM_RR_ with 20 iterations. (**g**) Normalized counts in the anterior wall of myocardium. (**h**) Normalized counts in the lateral wall of myocardium. (**i**) Normalized counts in the inferior wall of myocardium. (**j**) Normalized counts in the septal wall of myocardium. Solid and white bars denote CA and NCA, respectively. Error bars are SD of the mean. (***P < 0.001 and, ****P < 0.0001, Tukey-Kramer test in **f** and student’s *t* test in **g**–**j**). Abbreviations as in Fig. 1.

The polar maps of the anthropomorphic torso phantom were generated to confirm the difference between FBP and OSEM_RR_ in both CA and NCA. Polar maps derived from CA and NCA were visually similar in FBP and OSEM_RR_ (Fig. 3a,b). OSEM_RR_ with 10, 15, and 20 iterations yielded similar normalized count profiles in CA and NCA (Fig. 3c,d). A lower normalized count difference was exhibited in OSEM_RR_ with 10 iterations (−0.0061 ± 0.029), whereas higher normalized count differences were exhibited in FBP (0.039 ± 0.058) and OSEM_RR_ with 1 iteration (0.040 ± 0.037), (*p* < 0.0001, Fig. 3e,f). No significant differences between CA and NCA were exhibited in FBP and OSEM_RR_ with 1 and 5 iterations in the anterior wall (Fig. 3g), in FBP and OSEM_RR_ with 10, 15, and 20 iterations in the lateral wall (Fig. 3h), in OSEM_RR_ with 10, 15, and 20 iterations in the inferior wall (Fig. 3i), and in OSEM_RR_ with 1, 5,10, 15, and 20 iterations in the septal wall (Fig. 3j).

### Anthropomorphic torso phantom: myocardial defects model

**Figure 4:**
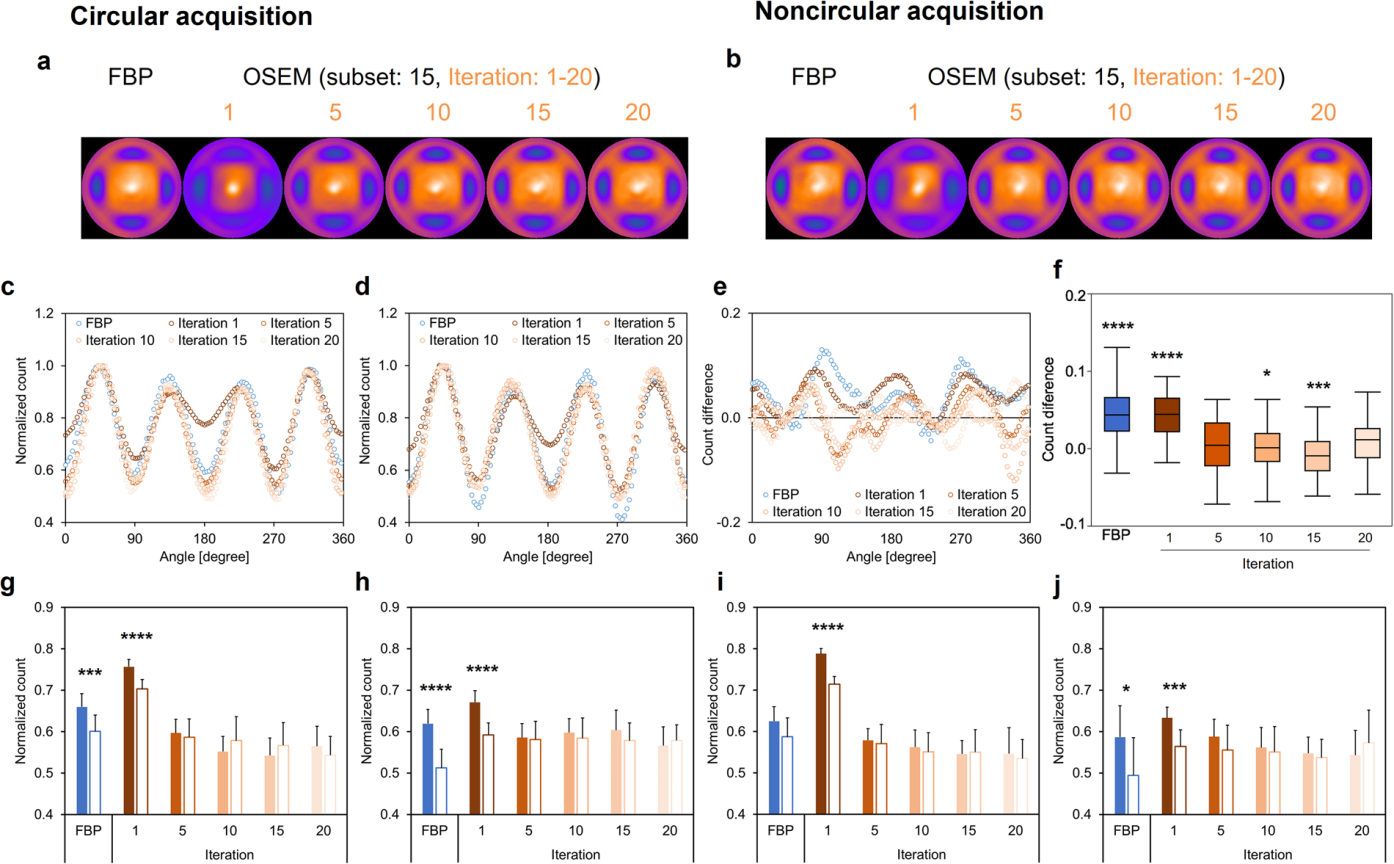
The anthropomorphic torso phantom images and its count distributions. Four plastic circular defects were placed in the mid-anterior, lateral, inferior, and septal walls. Polar maps were generated with FBP and OSEM_RR_ reconstructions in CA (**a**) and NCA (**b**). The iterations changed from 1 to 20, and a subset was constantly 15 in OSEM_RR_. (**c**) Normalized count distributions of the short axis image in CA. (**d**) Normalized count distributions of the short axis image in NCA. (**e**) Count differences between CA and NCA. (**f**) The box-and-whisker plot of the count differences. The count differences obtained from FBP and OSEM_RR_ with 1, 5, 10, and 15 iterations were compared with OSEM_RR_ with 20 iterations. (**g**) Normalized counts in the anterior defect of myocardium. (**h**) Normalized counts in the lateral defect of myocardium. (**i**) Normalized counts in the inferior defect of myocardium. (**j**) Normalized counts in the septal defect of myocardium. Solid and white bars denote CA and NCA, respectively. Error bars are SD of the mean. (*P < 0.05, ***P < 0.001, and ****P < 0.0001, Tukey-Kramer test in **f** and student’s *t* test in **g**–**j**). Abbreviations as in Fig. 1.

The polar maps of the anthropomorphic torso phantom with myocardial defects were generated to confirm the difference between FBP and OSEM_RR_ in both CA and NCA (Fig. 4a,b). FBP and OSEM_RR_ with 5, 10, 15, and 20 iterations yielded similar normalized count profiles in CA and NCA (Fig. 4c,d). Significant higher normalized count differences were exhibited in FBP (0.044 ± 0.038) and OSEM_RR_ with 1 iteration (0.043 ± 0.029) than in OSEM_RR_ with 20 iterations (0.009 ± 0.030) (*p* < 0.0001 for all, Fig. 4e,f). No significant differences between CA and NCA were exhibited in OSEM_RR_ with 5, 10, 15, and 20 iterations in the anterior (Fig. 4g), lateral (Fig. 4h), inferior (Fig. 4i), and septal walls (Fig. 4j). To confirm the compatibility of Gaussian and Butterworth filters, the phantom delineation created from Gaussian filter with 3.3-, 6.6-, 8.3-, 9.9-, 11.6-, 13.2-, 14.9-, and 16.5-mm FWHMs was compared with that from 0.64 Nyquist frequency of Butterworth filter in Supplementary Fig. S2. The smallest count difference was exhibited in the combination of Butterworth with 0.64 Nyquist frequency and Gaussian with 13.2-mm FHWM. OSEM_RR_ and FBP reconstructions with or without Gaussian filtering was also compared in Supplementary Fig. S3. The amount of statistical noise component without Gaussian filter was higher in FPB than in OSEM_RR_. Although the effect of Gaussian filter for the noise reduction was similar in FBP and OSEM_RR_ images, the SPECT count distribution for FBP significantly differed from that for OSEM_RR_ in the mid and basal regions.

### Clinical subjects

**Figure 5:**
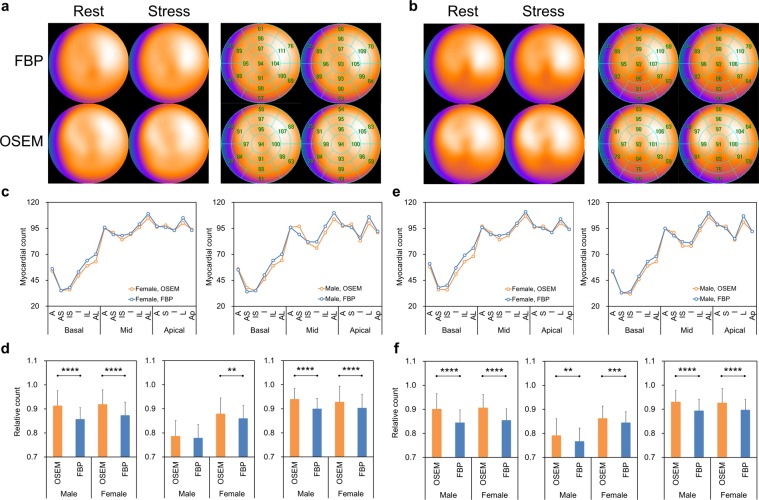
Myocardial perfusion distributions in clinical patients. (**a**) Polar maps created from 37 female patients with a low likelihood of CAD. The 17-segment polar maps were shown in the right panel. (**b**) Polar maps created from 35 male patients with a low likelihood of CAD. The 17-segment polar maps were also shown in the right panel. (**c**) Stress myocardial count distributions derived from FBP and OSEM_RR_ in male (right) and female (left) patients. (**d**) Relative count ratios of anterolateral segments to anteroseptal segments (left), inferior segments (middle), and anterior segments (right) in stress condition. (**e**) Rest myocardial count distributions derived from FBP and OSEM_RR_ in male (right) and female (left) patients. (**f**) Relative count ratios of anterolateral segments to anteroseptal segments (left), inferior segments (middle), and anterior segments (right) in rest condition. Error bars are SD of the mean. (**P < 0.01, ***P < 0.001, and ****P < 0.0001. Paired student’s *t* test). *A*, anterior; *AS*, anteroseptal; *S*, septal; *IS*, interoseptal; *I*, inferior; *IL*, inferolateral; *L*, lateral; and *AL*, anterolateral.

**Figure 6:**
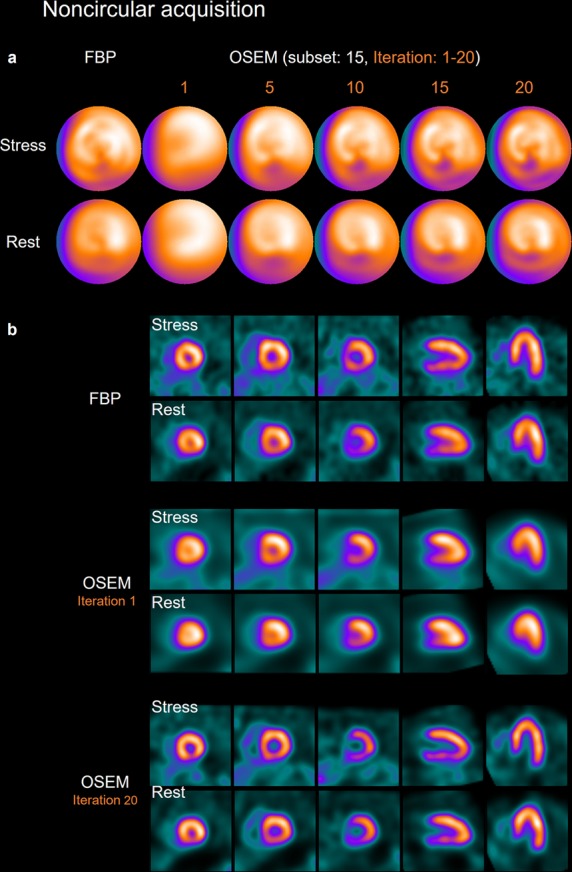
Clinical stress-rest polar maps (**a**) and SPECT images (**b**) in a 60-year-old male patient with slight ischemia in the inferior wall. Height and weight were 167 cm and 66 kg, respectively. All images were acquired with the noncircular orbit of gamma cameras. The Iterations changed from 1 to 20, and a subset was constantly 15.

The NDBs were generated to confirm the difference of clinical myocardial perfusion distribution between FBP and OSEM_RR_ in NCA (Fig. 5a,b). Myocardial perfusion counts were always high in the anterolateral region in both male and female NDBs. Moreover, those showed higher values in the anteroseptal region in OSEM_RR_. The segmental myocardial perfusion distributions derived from OSEM_RR_ paralleled those from FBP in both male and female NDBs (Fig. 5c,e). Based on count ratios of anterolateral segments to anteroseptal segments, inferior segments, and anterior segments (Fig. 5d,f), significantly higher myocardial perfusion counts were exhibited in OSEM_RR_ than in FBP. The clinical study demonstrated that slight ischemia was delineated in the inferior regions in FBP and OSEM_RR_ with 10, 15, and 20 iterations (Fig. 6a). The delineation of the LV cavity by OSEM_RR_ with 20 iterations was superior to that by FBP (Fig. 6b).

## Discussion

This present study showed that OSEM_RR_ could yield similar count distributions in CA and NCA in the phantom study. In the anthropomorphic torso phantom study with defects, defect delineation was equivalent between CA and NCA in OSEM_RR_ processing. Clinical myocardial perfusion showed a more uniform distribution in OSEM_RR_ than FBP in the NDB. Since the rotation radius varies greatly in SPECT acquisition, we would suggest the use of OSEM_RR_ in nuclear cardiology study. However, the OSEM_RR_ processing with 10, 15, and 20 iterations should need to yield superior myocardial perfusion.

Although our study revealed that OSEM_RR_ compensated for myocardial perfusion counts of NDBs in the anterior, anteroseptal, and inferior walls, uniform myocardial count distribution in the whole myocardium has not yet been shown in OSEM_RR_ processing. Even if attenuation and scatter corrections are used in MPI, uniform myocardial perfusion would not be observed^17,33^ because the anterior and inferior myocardial counts are influenced by respiratory motion owing to diaphragmatic movement in digital phantom studies^34–36^. Regarding the myocardial count distribution, maximum myocardium movement is observed in the apex^37^, and then myocardial ^99m^Tc distribution will be blurred. Consequently, further corrections for cardiac and respiratory movements are needed to find the true myocardial perfusion distribution.

The OSEM technology can easily be integrated with the RR algorithm. Furthermore, our results showed no disadvantages and artifacts owing to using OSEM_RR_ in the phantom scans and clinical NDBs. Although the FBP has commonly been used to reconstruct slices from projection images in nuclear cardiology, we highly recommend the use of OSEM_RR_ in daily clinical practice. Moreover, manufacturers have developed their own version of RR algorithms for OSEM iterative reconstruction, such as Astonish by Philips Medical Systems, Milpitas, CA, USA^38^, Evolution for Cardiac by GE Healthcare, Waukesha, WI, USA^39^, and wide beam reconstruction by UltraSPECT, Haifa, Israel^3,39^. Consequently, the optimal number of iterations and filtering parameters should need to be investigated by OSEM_RR_ fitted for each SPECT imaging system.

The effects of OSEM_RR_ in CA and NCA were evaluated in the cone-shape phantom, anthropomorphic torso phantom, and clinical patient studies. Although the orbit of the camera for the cone phantom experiment did not represent a clinical condition, our aim of this experiment was to confirm the SPECT count distribution of RR in SPECT acquisition using a simplified model. In the anthropomorphic torso phantom without defects, count distributions between FBP and OSEM seemed to be similar. However, defects delineation was different between CA and NCA in FBP. Myocardial perfusion counts of clinical NDB were corrected with OSEM_RR_ in the deep regions of the heart: anterior, anteroseptal, and inferior walls. The OSEM_RR_ processing could contribute to producing uniform myocardial perfusion counts even without the attenuation correction.

Our study has several limitations. We did not compare CA and NCA in the clinical study. No optimal reconstruction condition for the OSEM_RR_ was used in the clinical studies. However, a small number of OSEM iterations yielded insufficient compensation for the RR in the phantom and clinical studies (Figs. 2–4 and 6). Moreover, optimal smoothing filtering conditions for Gaussian and Butterworth filter was not evaluated. This is because OSEM_RR_ incorporating Butterworth filter is not supported by the Siemens data processing system. However, the effect of smoothing filtering condition, which was used in this study, has already been examined in our previous study^16^. Since quantitative scoring of myocardial perfusion using NDBs was not performed in the present clinical study, summed stress/rest/difference scores could be calculated to confirm the difference between OSEM_RR_ and FBP.

## Conclusion

We clarified the myocardial perfusion distribution corrected with RR in CA and NCA of SPECT imaging in the cone-shape and anthropomorphic torso phantom studies. The phantom study characterized the relationship between iterations and count distribution in OSEM_RR_ processing. Moreover, the count distribution derived from NCA paralleled those from CA. When NDBs were created to evaluate the myocardial perfusion distributions in clinical studies, OSEM_RR_ is recommended to compensate for the depth-dependent blurring in myocardial perfusion counts on the 17-segment polar map.

## Supplementary information

Supplementary Information 
