# Supplementary material for: Impact of iterative reconstruction with resolution recovery in myocardial perfusion SPECT: phantom and clinical studies
Source: Sci Rep. 2019 Dec 23;9:19618. doi: 10.1038/s41598-019-56097-4 (PMC6928019; doi:10.1038/s41598-019-56097-4)
Supplement: Supplementary file 1 — Supplementary Information [file 41598_2019_56097_MOESM1_ESM.pdf]

Supplementary information

Impact of iterative reconstruction with resolution recovery in myocardial perfusion SPECT: phantom and clinical studies

Koichi Okuda, Kenichi Nakajima, Hiroto Yoneyama, Takayuki Shibutani,

Masahisa Onoguchi, Shinro Matsuo, Mitsumasa Hashimoto, Seigo Kinuya

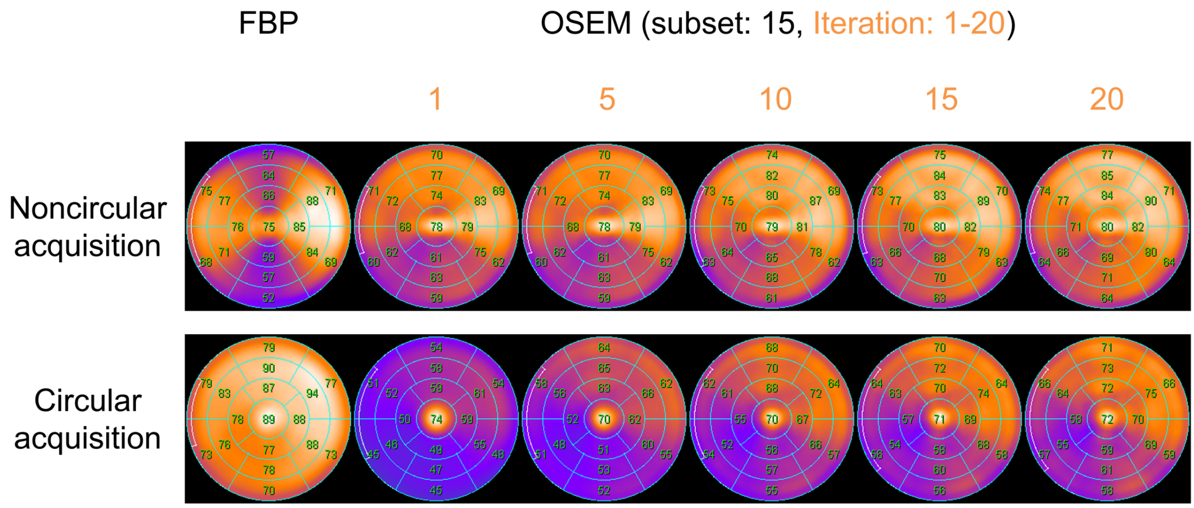

Supplementary Figure S1. The cone phantom images derived from noncircular acquisition and circular acquisition. Polar maps were generated with FBP and $OSEM_{RR}$ reconstructions and segmented into 17 regions.

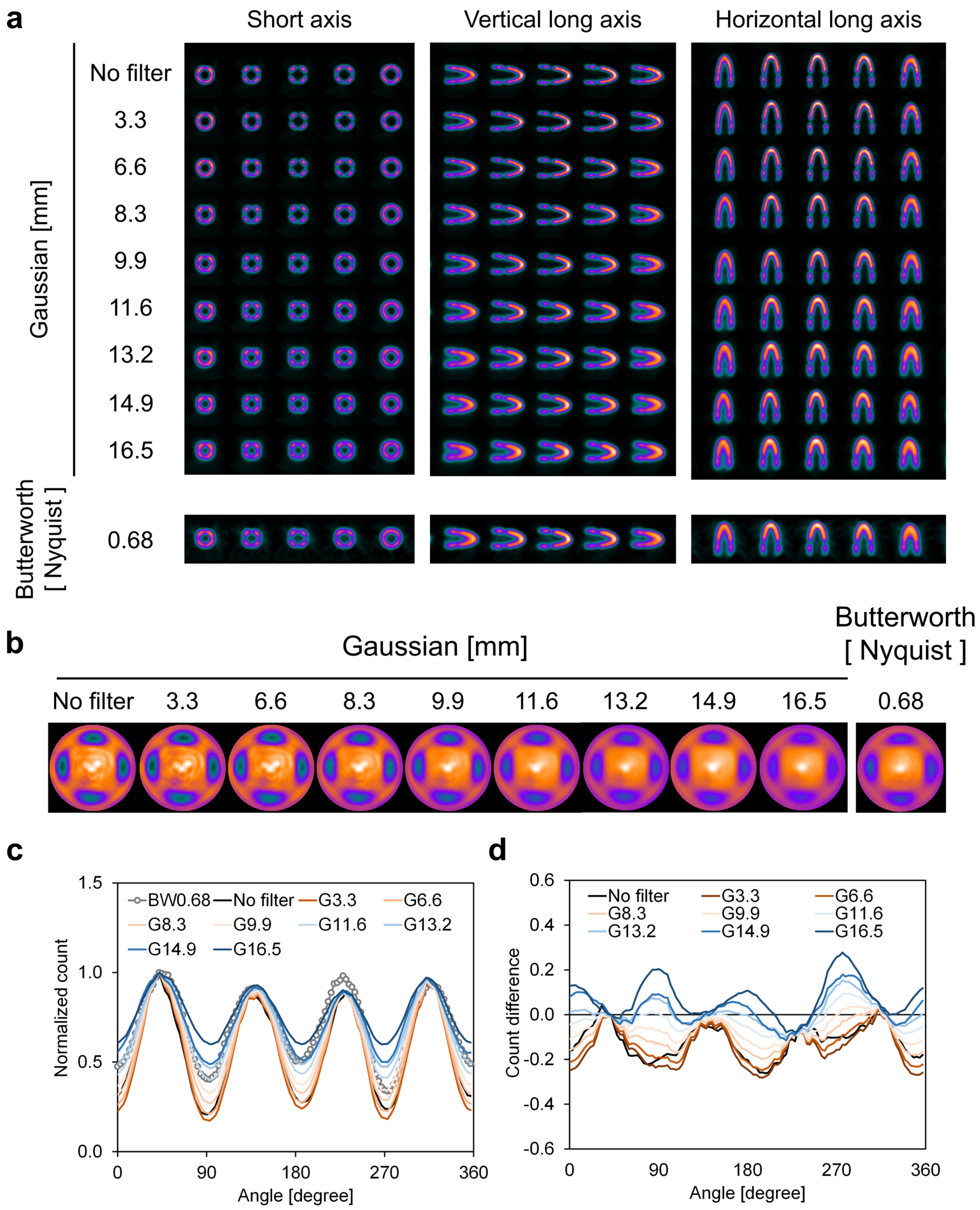

Supplementary Figure S2. The compatibility assessment of Gaussian and Butterworth filters. (**a**) Short axis, vertical long axis, and horizontal long axis display of anthropomorphic torso phantom with defects. The $OSEM_{RR}$ with Gaussian filter and FBP with Butterworth filter were used, and the iterations and subsets were 10 and 15 in OSEM processing, respectively. The Gaussian filters with 3.3-, 6.6-, 8.3-, 9.9-, 11.6-,

13.2-, 14.9-, and 16.5-mm FWHMs were applied in the $OSEM_{RR}$ processing. Our clinical filtering condition was 0.68 Nyquist frequency of Butterworth filter. (**b**) Polar map display of anthropomorphic torso phantom with defects. (**c**) Normalized count distributions of the short axis images derived from FBP with Butterworth filter of 0.68 Nyquist frequency and $OSEM_{RR}$ with Gaussian filters of 3.3-, 6.6-, 8.3-, 9.9-, 11.6-, 13.2-, 14.9-, and 16.5-mm FWHMs. (**d**) Count distributions for $OSEM_{RR}$ with Gaussian filter subtracted from those for FBP with Butterworth filter. Mean count differences for $OSEM_{RR}$ with no filtering; Gaussian filters with 3.3- (G3.3); 6.6- (G6.6); 8.3- (G8.3); 9.9- (G9.9); 11.6- (G11.6); 13.2- (G13.2); 14.9- (G14.9); and 16.5-mm (G16.5) FWHMs were -0.12 ± 0.074, -0.15 ± 0.082, -0.13 ± 0.072, -0.093 ± 0.060, -0.063 ± 0.052, -0.026 ± 0.051, 0.0060 ± 0.057, 0.026 ± 0.069, and 0.072 ± 0.084, respectively.

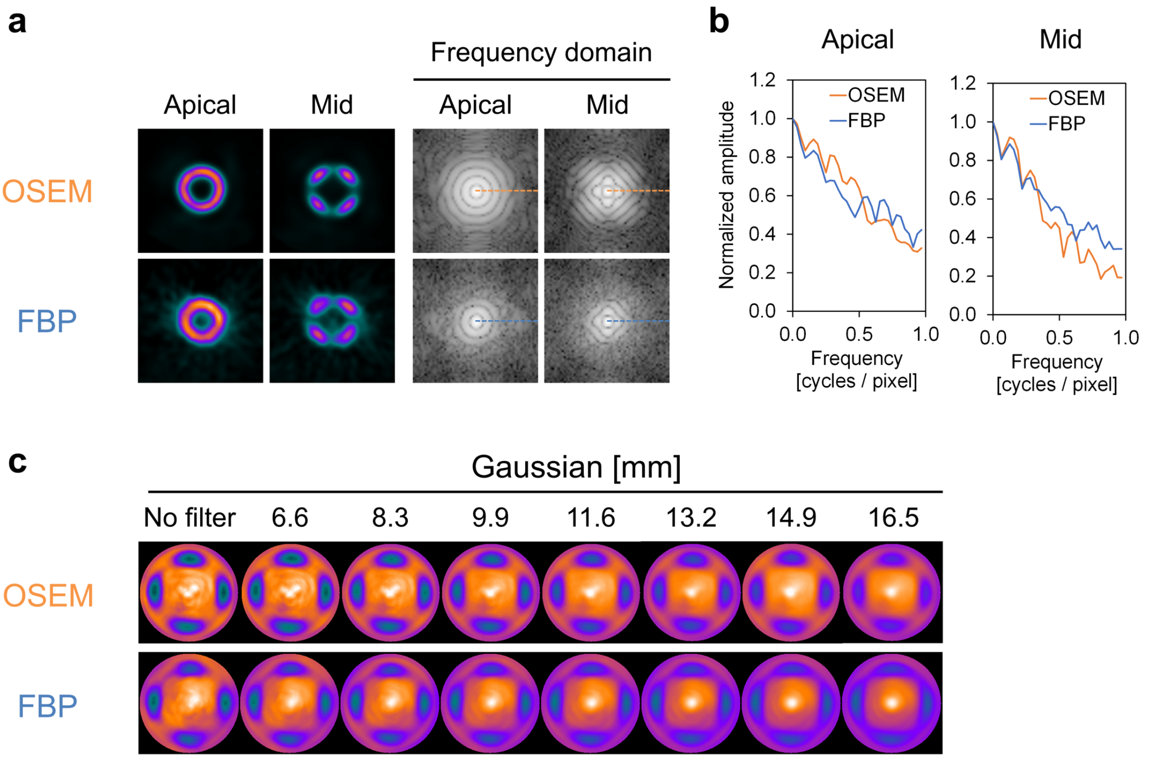

Supplementary Figure S3. Comparison of $OSEM_{RR}$ and FBP methods with Gaussian filter in the anthropomorphic torso phantom study. (**a**) Apical and mid short axis images and frequency domain images. Gaussian filter was not used in both FBP and $OSEM_{RR}$. The iterations and subsets were 10 and 15 in OSEM processing, respectively. (**b**) Fast Fourier transform plots of the $OSEM_{RR}$ and FBP images in the apical and mid slices. (**c**) Polar map displays of $OSEM_{RR}$ and FBP with or without Gaussian filter of 6.6-, 8.3-, 9.9-, 11.6-, 13.2-, 14.9-, and 16.5-mm FWHMs. Gaussian filter was used as post-reconstruction filtering in both reconstructions.
